# Induction of Apoptosis and Reduction of Endogenous Glutathione Level by the Ethyl-Acetate Soluble Fraction of the Methanol Extract of the Roots of *Potentilla fulgens* in Cancer Cells

**DOI:** 10.1371/journal.pone.0135890

**Published:** 2015-08-18

**Authors:** Debabrata Tripathy, Alka Choudhary, Uttam Chand Banerjee, Inder Pal Singh, Anupam Chatterjee

**Affiliations:** 1 Molecular Genetics Laboratory, Department of Biotechnology & Bioinformatics, North-Eastern Hill University, Shillong, Meghalaya-793022, India; 2 Department of Pharmaceutical Technology Biotechnology, National Institute of Pharmaceutical Education and Research, Sector-67, S.A.S. Nagar, 160062, Punjab, India; 3 Department of Natural Products, National Institute of Pharmaceutical Education and Research, Sector-67, S.A.S. Nagar, 160062, Punjab, India; National Cheng Kung University, TAIWAN

## Abstract

*Potentilla fulgens* root traditionally used as a folk remedy in Meghalaya, India. However, systematic evaluation of its anticancer efficacy was limited. We investigated the anticancer potentials of the various extracts prepared by partitioning of the methanol extract of the root with the aim to discover major contributing factors from the most effective fractions. Methanol extract of *P*. *fulgens* roots (PRE) was prepared by maceration which was subsequently fractionated into hexane, ethyl-acetate (EA) and *n*-butanol soluble fractions. Various assays (clonogenic assay, Flow cytometry analysis, western blot, semiquantitative RT-PCR and the level of endogenous glutathione) were used to evaluate different parameters, such as Cell survivability, PARP-1 proteolysis, expression pattern of anti-apoptotic and γ-glutamyl-cysteine synthetase heavy subunit (GCSC) genes in both MCF-7 and U87 cancer cell lines. Since the EA-fraction showed most efficient growth inhibitory effect, it was further purified and a total of nine compounds and some monomeric and dimeric flavan-3-ols were identified and characterized. Three compounds viz., epicatechin (EC), gallic acid (GA) and ursolic acid (UA) were taken on the basis of their higher yield and 10 μg/ml of each was mixed together. The concentration used in this study for PRE, EA- and Hex-fraction was 100 μg/ml, which was higher than the IC_50_ value. Apoptotic cell death in the PRE, EA-fraction and EC+GA+UA treated cancer cell cultures was significantly greater than in normal cells due to suppression of anti-apoptotic protein Bcl2 following treatment. Depletion of glutathione by downregulating GCSC was also observed. Induction of apoptosis and lowering the level of glutathione are considered to be positive activity for an anticancer agent. Therefore, modulation of GSH concentration in tumor cells by PRE and its EA-fraction opened up the possibility of a new therapeutic approach because these plant products are not harmful to normal cells and may regulate the tumor cellular response to different anticancer treatments. Thus, it would be interesting to examine efficacy of these plant products or EA-fraction in human cancer treatment.

## Introduction

Traditional medicinal plants as potential cancer preventive and therapeutic agents have received increasing attention during the past years [1,2]. The genus *Potentilla* belongs to family *Rosaceae* which includes about 500 species, and is mainly distributed in temperate, arctic and alpine zones of the Northern hemisphere [3]. *Potentilla* extracts are considered to be safe and non-toxic when applied to humans [4,5]. Extracts from the root of *Potentilla anserina* have been used for the treatment of certain viral infections as folk medicinal herbs in Tibet [6]. It was proposed that the presence of higher amount of hydrolysable and condensed tannins, flavonoids and triterpenes in most plant parts of *Potentilla* species could be an important factor for the observed biological effects [7].

*Potentilla fulgens* Wall. Ex Hook, commonly known as Himalayan Cinquefoil in English, is an important medicinal plant of higher reaches of the Himalaya. Different ethnic groups of North-East India use different plant parts as a source of medicine, though their mode of action is yet to be established. Inhabitants of this region traditionally chew betel-nut (*Areca catechu*), betel leaves (*Piper betel*) and the tap root of *P*. *fulgens* for various ailments [8] but despite its extensive use little is known about its phytochemistry and mechanism of action. Earlier studies on methanol extract of the root of *P*. *fulgens* have shown better survival of the mice bearing Ehrlich ascites cells, and also a dose-dependent inhibitory effect on growth of MCF-7 cells [9]. A recent attempt to isolate and characterize pure compounds from the ethyl-acetate soluble fraction of the methanol extract of the roots of *P*. *fulgens* have yielded nine compounds including two new ursane type triterpenoids Fulgic acid A and Fulgic acid B. Both these new compounds show good antioxidant activity [10]. More extensive studies using various analytical techniques have established that flavans including oligomeric flavanols followed by triterpene acids are the major constituents of the root of *P*. *fulgens* [11].

The present study aims to investigate the effects of different fractions of *P*. *fulgens* root extract on cell survival, cell proliferation, apoptosis and endogenous GSH-level in mammalian cancer cells and to determine the major contributing factors for such effect from the most effective fraction of this root extract. We demonstrate that *P*. *fulgens* root extract and its ethylacetate soluble fraction inhibit the growth of cancer cells by inducing apoptosis. We have also analyzed the status of endogenous glutathione (GSH) in the treated cancer cells and shown its depletion, considered to be a positive effect of any chemotherapeutic drug because lowering the level of endogenous GSH makes the cell more sensitive to drug [12]. Since GSH was found to play an important role in cell death regulation and its depletion requires for the execution of apoptosis [13], therefore effort to develop anticancer drugs targeting the redox systems, for example, glutathione and thioredoxin, have attracted attention.

## Materials and Methods

### Plant Material

Roots of *P*. *fulgens*, a non-endangered plant, were collected from 20 different plants at Shillong peak forest area (altitude 1700 m above sea level; 25°34 North latitude and 91°54 East longitude) of Meghalaya state of India after obtaining a proper approval of the forest officer. A voucher specimen was deposited in the herbarium of the Department of Botany, North-Eastern Hill University, Shillong (accession number 11906). The root material (1 kg) was air dried under shade for a week and ground in a mixer grinder to a coarse powder.

### Extraction and isolation

**Fig 1:**
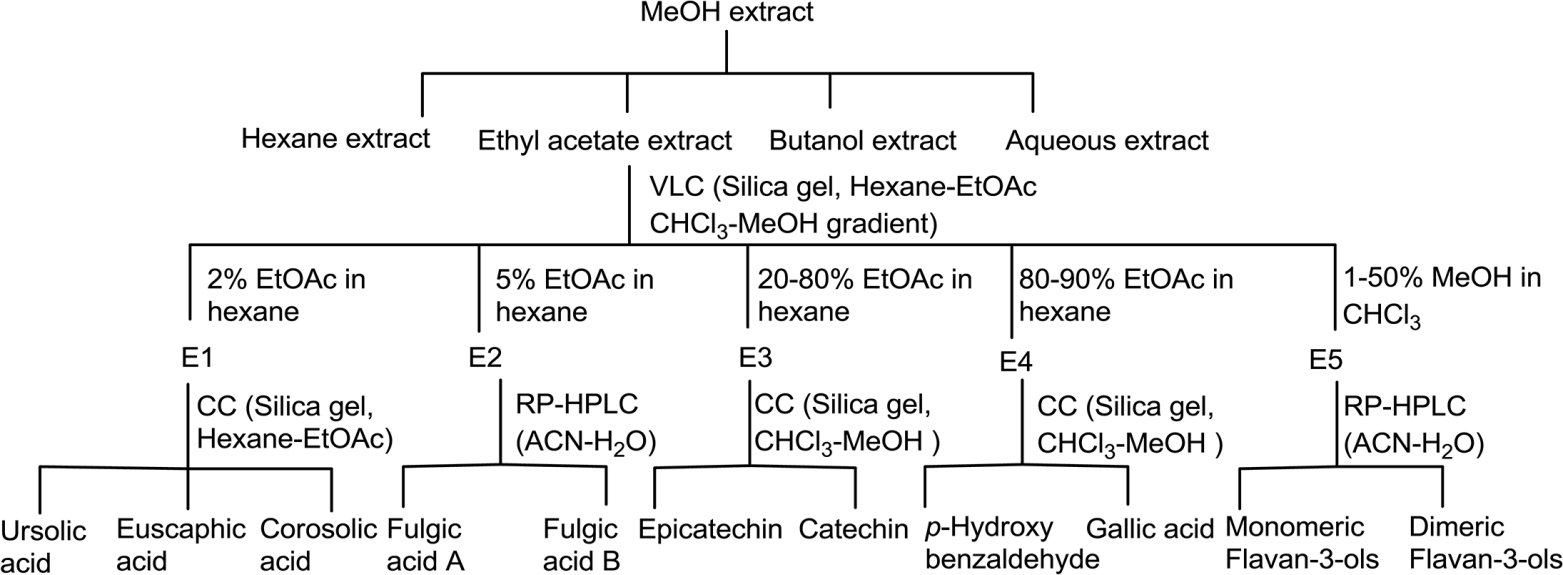
Flow diagram of Extraction and Isolation of chemical constituents from *P*. *fulgens* roots.

Ursolic acid, euscaphic acid, corosolic acid, fulgic acid A and B, epicatechin, catechin, gallic acid, p-hydroxybenzaldehyde along with several dimeric flavan-3-ols were isolated as described in our previous communications [10]. Briefly, methanol extract (MeOH) (80:20) of *P*. *fulgens* roots (PRE) was prepared by maceration which was subsequently fractionated into hexane (Hex), ethyl-acetate (EA) and *n*-butanol soluble fractions. The ethyl-acetate extract was subjected to vacuum liquid chromatography using hexane-ethyl acetate and chloroform-methanol gradients to yield five pooled fractions, E1 to E5. Column chromatography of fraction E1 yielded ursolic acid, euscaphic acid and corosolic acid. Two stereoisomeric triterpene acids, fulgic acid A and fulgic acid B were separated from fraction E2 by HPLC. Fractions E3 to E5 after column chromatography and reverse phase HPLC yielded phenolics catechin, epicatechin, gallic acid, p-hydroxybenzoic acid, various monomeric and dimeric flavan-3-ols [10,11]. Isolation scheme is shown in Fig 1.

#### Cell line and clonogenic cell survival assay

MCF-7 (human breast cancer cell line) and U87 (human malignant glioma cell line) were purchased from the National Centre for Cell Science (Pune, India). Cells were cultured in Dulbecco’s MEM medium (DMEM, Invitrogen-Gibco) supplemented with 10% Fetal Calf serum (Invitrogen-Gibco), 100 u/ml Penicillin and 100 mg/ml Streptomycin (Invitrogen-Gibco) and 2 mM L-Glutamine (Invitrogen-Gibco).

The cell survivality was evaluated using a clonogenic assay in both the cancer cell lines. Briefly, cells were trypsinized and appropriate cell numbers (2000 and 3000 cells) were seeded into three 25 cm^2^ flasks each and for untreated controls, four flasks were plated at one cell density (1000 cells). Five hours after seeding, the cells were exposed for 24 h with 5 to 150 μg/ml of the root extract (PRE) of *P*. *fulgens* or 10 to 120 μg/ml of EA- or Hex- or *n*butanol-fraction. After the treatment cells were washed twice with the medium and finally flasks were incubated in a humidified incubator with 5% CO_2_ at 37°C with fresh Dulbecco’s MEM medium with 10% foetal calf serum for 10 days. The colonies were fixed and stained with 0.2% crystal violet in 70% ethanol. Each assay was performed in triplicate and colonies containing at least 50 cells were counted. The number of surviving colonies (treated samples) was normalized against the number of colonies in untreated samples.

### Treatment details

In all the experiments that were carried out after colonogenic assay, cancer cells were treated with 100 μg/ml of PRE, EA-fraction and Hex-fraction of the methanol extract of the roots of *P*. *fulgens*. In case of mixture of three compounds which were isolated from the EA-fraction i.e epicatechin (EC), ursolic acid (UA) and gallic acid (GA), each was taken 10 μg/ml. These three compounds were taken from three different EA-subfractions (fraction 1, 3 and 4) and their selection was based on their higher yield. In all these experiments the cells were exposed for 24h.

### Cell killing ability by PRE and EA-fraction to normal and cancer cells

This was determined by Trypan blue exclusion assay. Freshly collected human peripheral blood lymphocytes was used as normal cells and MCF-7 and U87 cells were used as cancer cells.

Heparinized peripheral blood from two healthy male donors (HPBL) was obtained after having their written consent for participation and used soon after its collection. Peripheral blood mononuclear cells were isolated by Ficoll-Hypaque (Sigma Diagnostics, St. Louis, MO) density gradient centrifugation (specific gravity 1.077 g/ml) for 30 min at 400*g* from these freshly drawn heparinized whole blood. Lymphocytes cultures were set up in RPMI 1640 medium supplemented with 10% heat inactivated FCS. Penicillin (100 U/ml) and streptomycin (100 mg/ml) and 2 mM L-Glutamine were added to the medium. Lymphocytes were stimulated with PHA and after 24 h lymphocytes were treated with PRE and EA-fraction (100 and 150 μg/ml) for 24 h. These cultures were incubated at 37° C and were harvested soon after the treatment. This study was approved by the Institutional and Human Ethics Committee. In case of cancer cells, MCF-7 and U87 cells were used and treated with PRE and EA-fraction (100 and 150 μg/ml) for 24 h. Cells were washed with fresh medium and then incubated for 10 min at room temperature with 0.4% final trypan blue. Dead cells were stained blue, and live ones were unstained. Experiments were repeated three times.

Cell killing ability by PRE and EA-fraction was also evaluated by flow-cytometric analysis (S1 File).

### Effect on cell proliferation in MCF-7 and U87 cells

The cellular proliferation assay was performed in MCF-7 and U87 cells after treating the cells with PRE, EA and Hex-fractions. Cells at a density of 5 x 10^5^ were grown in 25 cm^2^ flasks in DMEM medium at 37°C and 5% CO_2_. After 24 h of seeding, the cells were exposed to PRE, EA or Hex-fraction at a final concentration of 100 μg/ml for 24 h and finally fixed in 70% ethanol.

All the above fixed cells were washed in PBS and resuspended in 500 μl of propidium iodide solution (20 μg/ml propidium iodide, 0.2 mg/ml RNase) for 1 h incubation at room temperature in the dark. 10,000 cells were acquired for each sample and analyzed them in a FACS Calibur (Becton-Dickinson) using Cellquest software in order to quantify cell cycle compartments to estimate the percentage of cells distributed in the different cell cycle phases.

### Flow cytometric analysis of mitochondrial membrane potential

The dissipation of the mitochondrial membrane potential is considered to be a mitochondrial disruption event before apoptosis. It is caused by a sudden increase in the inner mitochondrial membrane permeability, known as the mitochondrial permeability transition [14]. Mitochondrial damage was evaluated using the lipophilic fluorescent probe 5,5′,6,6′-tetrachloro-1,1′,3,3′-tetra-ethyl-benzimidazol-carbocyanine iodide according to the manufacturer’s protocol (JC-1; BD Mitoscreen JC-1 kit; Cat 51302). Apoptotic cell death was evaluated in MCF-7 and U87 cells treated with and without PRE, EA-fraction, Hex-fraction and mixture of EC, UA and GA using JC-1 stain. In brief, JC-1 working solution was prepared in 1xAssay buffer and added 0.5 ml JC-1 stain to 1x10^6^ MCF-7 or U87 cells for 10 min at 37°C in CO_2_ incubator. The cells were washed twice with 1x Assay buffer at room temperature and finally JC-1 fluorescence was measured using a Becton Dickinson FACScalibur analytical flow cytometer (BD Biosciences, San Jose, CA). The percentage of cells of green (530 nm) and red (590 nm) fluorescence of JC-1 was analyzed.

### Immunoblotting

Immunoblot analyses were performed to detect the expression of PARP and β-actin proteins in untreated and treated MCF-7 and U87 cells. Cells were plated in 25 cm^2^ flasks at a concentration of 4 x 10^5^ and 20 h after they were exposed to PRE, EA, Hex and mixture of EC+GA+UA for 24 h. Cells were collected soon after the treatment.

All the collected cells were washed with PBS and proteins were extracted in cell lysis buffer (50mM Tris (pH 8.0), 150mM Nacl, 10% glycerol, 1% NP–40, 0.5% Sodium deoxycholate, 0.1% SDS and 0.42% NaF) containing protease inhibitor (1mM phenylmethylsulfonyl and 100 U/ml aprotinin). Protein concentrations were determined using the bicinchonic acid protein assay and 40 μg protein lysates from each sample was loaded in Novex Tris-Glycine 4–20% gradiaent gels and electrophoresis was performed in NuPAGE electrophoresis system from (Invitrogen, USA). Proteins were transferred to PVDF membranes (Sigma) following the standard protocol. The membranes were probed with a 1:1000 dilution of a rabbit polyclonal antibody against PARP Ab-3 (Neo-Markers, USA) and mouse monoclonal antibody against β-actin (AC-15; ab6276; abcam, USA). The membranes were blocked in 5% non-fat dried milk, freshly made in TBST (10mM Tris-Cl, 150mM NaCl, 0.05% Tween 20) buffer. Alkaline-phosphatase conjugated anti-mouse IgG (Abcam, USA) used as secondary antibody, and immunodetection was obtained by treating the blot with the substrate solution of BCIP/NBT (Bangalore Genei, India). The whole experiment was repeated twice.

### DNA fragmentation assay

Fragmentation of DNA as an indication of apoptosis is a commonly used assay in drug-cell interaction studies. The MCF7 and U87 cells (5 × 10^5^ cells/flask) were cultured for 24 h and then exposed to PRE, EA or Hex-fraction at a final concentration of 100 μg/ml for 24 h. Cells were washed once with ice-cold PBS, and resuspended in 250 μl lysis buffer (10 mM Tris–HCl, pH7.6, 20 mM EDTA, pH 8.0 and 0.5% (w/v) Triton X-100). After centrifugation at 12,000 rpm for 5 min, the supernatant was extracted once with phenol/chloroform (1:1) and once with chloroform / isoamyl alcohol (24:1). DNA was precipitated with sodium acetate (pH 5.2) at −20°C overnight. The DNA was then pelleted and subsequently digested with DNase-free RNAase (Amersham Biosciences UK Ltd., Little Chalfont, Buckinghamshire, UK) at 37°C for 20 min. Electrophoresis was carried out in a 2.0% (w/v) agarose gel and was examined under UV transillumination following ethidium bromide staining to determine the extent of apoptotic DNA fragmentation.

### Semiquantitative RT-PCR (reverse transcription polymerase chain reaction)

The expression of Bcl2, Survivin, cIAP, XIAP, GCLC (glutamate-cysteine ligase catalytic subunit) and GAPDH was analyzed in MCF-7 and U87 cells after the treatment with PRE, EA, Hex and mixture of EC+GA+UA. Total RNA was extracted from cells using RNAeasy kit (Qiagen GmbH, Hilden Germany). cDNAs were reverse transcribed from 1 μg of total RNA from each sample using Quantiscript Reverse Transcriptase, Quantiscript-RT-buffer and RT-primer-mix of QuantiTect Reverse Transcription kit (Qiagen GmbH, Hilden Germany) according to the manufacturer’s protocol.

Amplification of cDNA was carried out in 20 μl solution containing 2 μl cDNA, 10 pmol primer pairs for Bcl2, Survivin, XIAP, cIAP and GAPDH (for primer sequences- Table A in S1 File) and 10 μl of RT qPCR Master mix (Qiagen GmbH, Hilden, Germany). The PCR consisted of initial denaturation at 94°C for 5 min, followed by 25 reaction cycles (30 seconds at 94°C, 30 seconds at 60°C, and 30 seconds at 72°C) and a final cycle at 72°C for 10 min. GAPDH was used as internal control. The amplified PCR products were separated by agarose gel electrophoresis and visualized with ethydium bromide. The abundance of each target mRNA was detected and normalized to that of GAPDH mRNA.

### Determination of GSH level

Total cellular GSH content was estimated by the method of Akerboom and Sies [15] in MCF-7 and U87 cells after treatment with PRE, EA, Hex and EC+GA+UA. Cells were seeded in 25 cm^2^ flasks at a concentration of 5 x 10^4^ and when the cells reached confluency between 75–80%, the PRE or EA-fraction or Hex-fraction or EC+GA+UA was added for 24 h. Both treated and untreated cells were flashed into ice-cold 0.1 M phosphate buffered saline solution (pH 7.4), and the volume was made up to 1ml. Cells were counted in a haemocytometer and processed for determination of total GSH level as described earlier [16]. Briefly, after deproteinization by 10% ice-cold 5-sulfosalicylic acid 190 μl sample suspension was taken and added to 700 μl NADPH (0.3 mM), 100 μl DTNB (6 mM) and 10 μl GSH reductase (6 units/ml) and the optical density of the samples was measured at 412 nm using the UV-visible spectrophotometer (Cecil series 1000, Camlab, UK). The GSH concentrations were determined by comparison with standards.

### Statistics

The results are expressed as mean plus or minus Standard deviation (SD). Statistical analysis of the data was performed by paired t-test and p values <0.05 were considered significant.

The data obtained from clonogenic survival assay are represented as a sigmoidal fit curve with a linear X scale, the function used was Boltzmann. The Y value at X_0_ is considered to be half way between the two limiting values on Y-axis and this is considered to be the inhibitor concentration 50 (IC_50_) value.

## Results

### Effect on clonogenic efficiency

**Fig 2:**
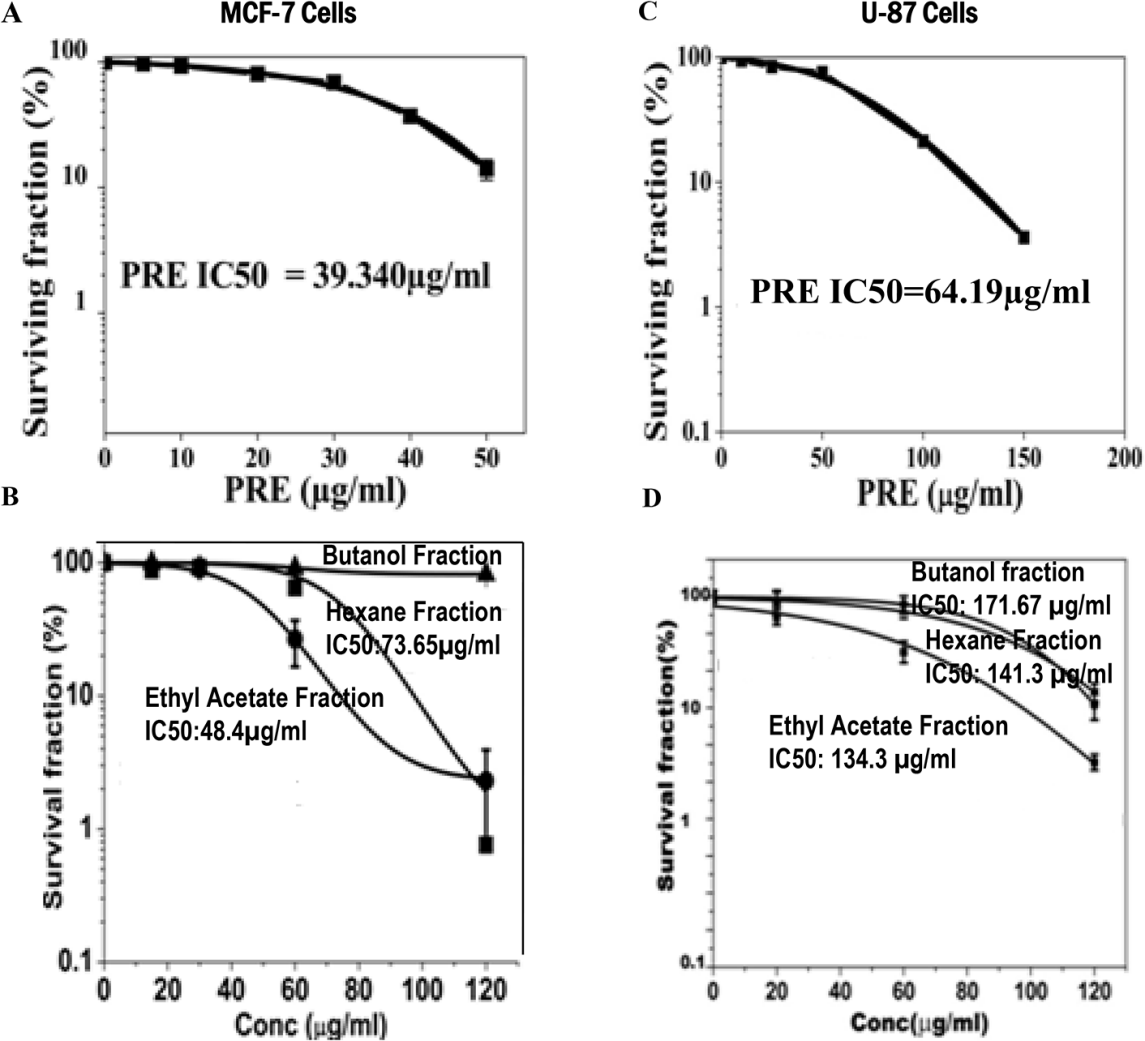
Cells were treated for 24 h with various concentrations of crude extract of *P*. *fulgens* root (PRE) and its ethyl acetate, hexane and *n*butanol soluble fractions. After the indicated time of treatment, cells were maintained without test materials for 10 days. At day 10, the grown colonies were counted and percentage of survival fraction was then calculated and shown in **(A and B)** in MCF-7 cells and **(C and D)** in U87 cells. Results were expressed as means ± SD for three independent determinations.

MCF-7 and U87 cells were treated with different concentrations of PRE, and EA-, Hex- and butane-extracted fractions. Whereas PRE, EA-fraction and Hex-fraction caused dose dependent reduction in the cloning efficiency in both cell lines (Fig 2A–2D) the butanol-fraction did not do so (Fig 2B). However, the degree of reduction in cloning efficiencies in both the cell lines was maximum with PRE. The value of inhibition concentration (IC_50_) deduced from the Sigmoidal fit graph was 39.34 μg/ml with PRE whereas for EA it was 48.4 μg/ml in MCF-7 cells. For U87, these values were 64.2 and 134.3 μg/ml (Fig 2A) with PRE and EA-fraction, respectively.

### PRE and EA-fraction kill more cancer cells than normal cells

The frequencies of dead cells are more in the cancer cells than normal lymphocytes after treatment with PRE or EA-fraction (Table 1). Significant increase in sub-G1 cells in the cancer cell lines (measured by FACS) relative to the normal lymphocytes after the treatment with PRE or EA-fraction also point to the increased apoptosis in MCF-7 and U87 (Fig A in S1 File).

**Table 1:** Trypan Blue Exclusion Assay in human lymphocytes and in cancer cell lines after treatment with PRE or EA-fraction.

Cell Type	Percentage of Dead cells ±SD
**Primary Normal Cell**	
Untreated Lymphocytes	8.8±0.8
PRE (**100μg/ml**) treated	18.8±2.3 p = 0.06
PRE (**150μg/ml**) treated	24.6±2.2^**a**^
EA (**100μg/ml**) treated	14.2±1.4 p = 0.07
EA (**150μg/ml**) treated	22.9±2.2 ^**a**^
**Cancer Cell lines**	
Untreated MCF-7 cells	5.5±0.9
PRE (**100μg/ml**) treated	49.1±1.8 ^**a**^ p = 0.009
PRE (**150μg/ml**) treated	52.4±2.4 ^**a**^
EA (**100μg/ml**) treated	38.6±2.5 ^**a**^ p = 0.02
EA (**150μg/ml**) treated	42.9±2.2 ^**a**^
Untreated U87 cells	4.9±0.8
PRE (**100μg/ml**) treated	42.6±2.5 ^**a**^ p = 0.02
PRE (**150μg/ml**) treated	48.4±2.4 ^**a**^
EA (**100μg/ml**) treated	29.1±1.7 ^**a**^ p = 0.01
EA (**150μg/ml**) treated	33.9±2.2 ^**a**^

Student’s t-test with respect to respective control value.

^**a**^ Significant in Paired t-test.

### Flow cytometric analysis of cells after treatment with the extract

**Fig 3:**
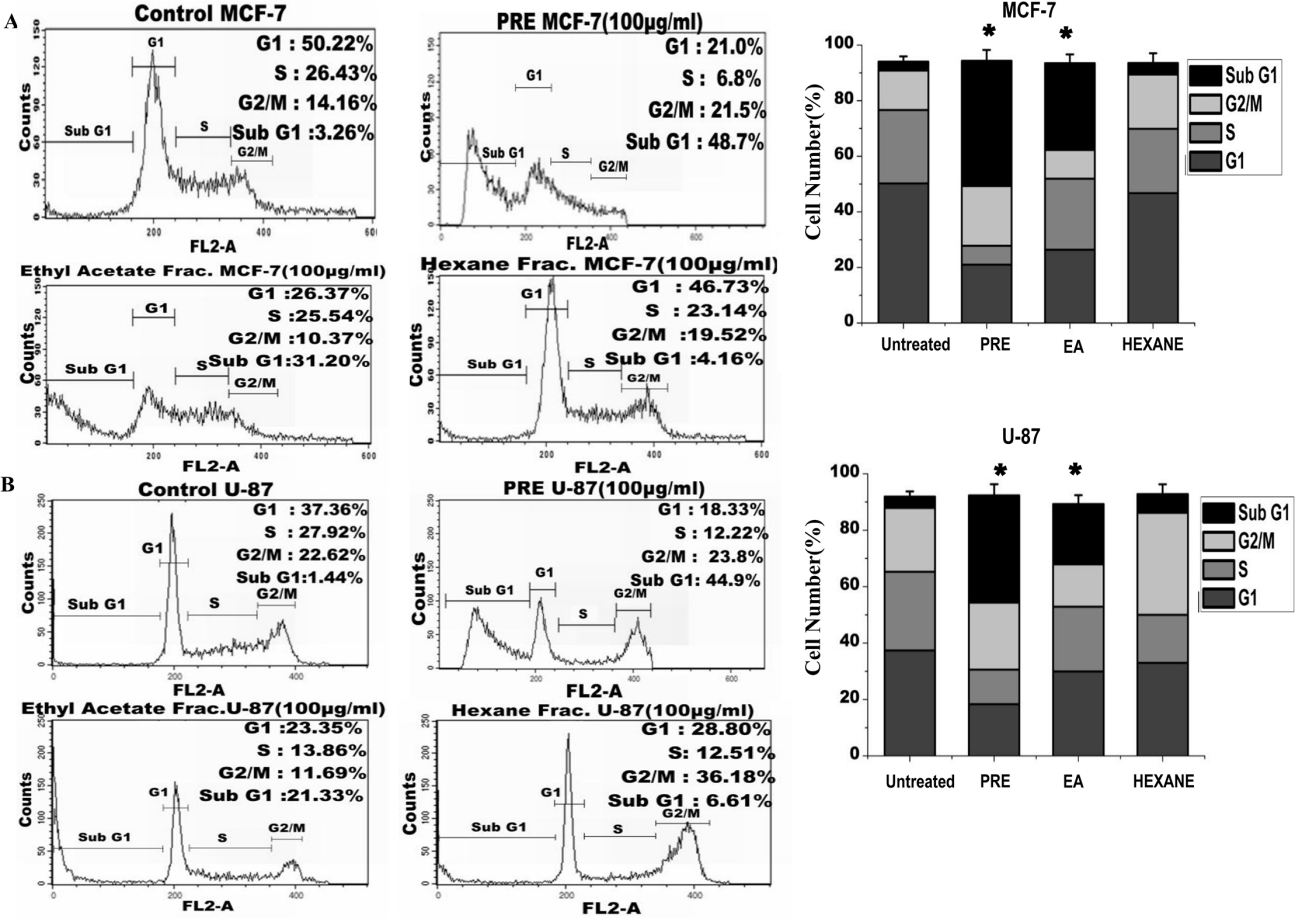
Analysis of cell cycle (A) The flow cytometry analysis on cell cycle distribution of PI-labeled MCF-7 cells and (B) in U87 cells with and without treatment with PRE, ethyl acetate (EA) and hexane (Hex) soluble fractions of *P*. *fulgens* root extract. Right panel-The percentage of cells at different stages in both MCF7 and U87 cells with and without treatment. The values are the mean±SEM of three independent experiments. *p<0.05, Students’t-test as compared with untreated control.

The distribution of cells in different phases of cell cycle was determined by flow cytometric analysis of DNA content in MCF-7 (Fig 3A) and U87 cells (Fig 3B). As shown in the figure, no significant accumulation of cells in any phase was observed in any of the treated cell lines. However the increased fraction of cells in sub-G1 phase was observed in samples treated with PRE (49% in MCF-7 and 45% in U87 cells) and EA-fraction (MCF-7: 31%, U87: 21%). The right panel of the Fig 3A and 3B showed significant increase in the frequency of sub-G1 cells after the treatment with PRE and EA-fraction. Hexane-fraction did not alter the frequency in sub-G1 phase cells.

**Fig 4:**
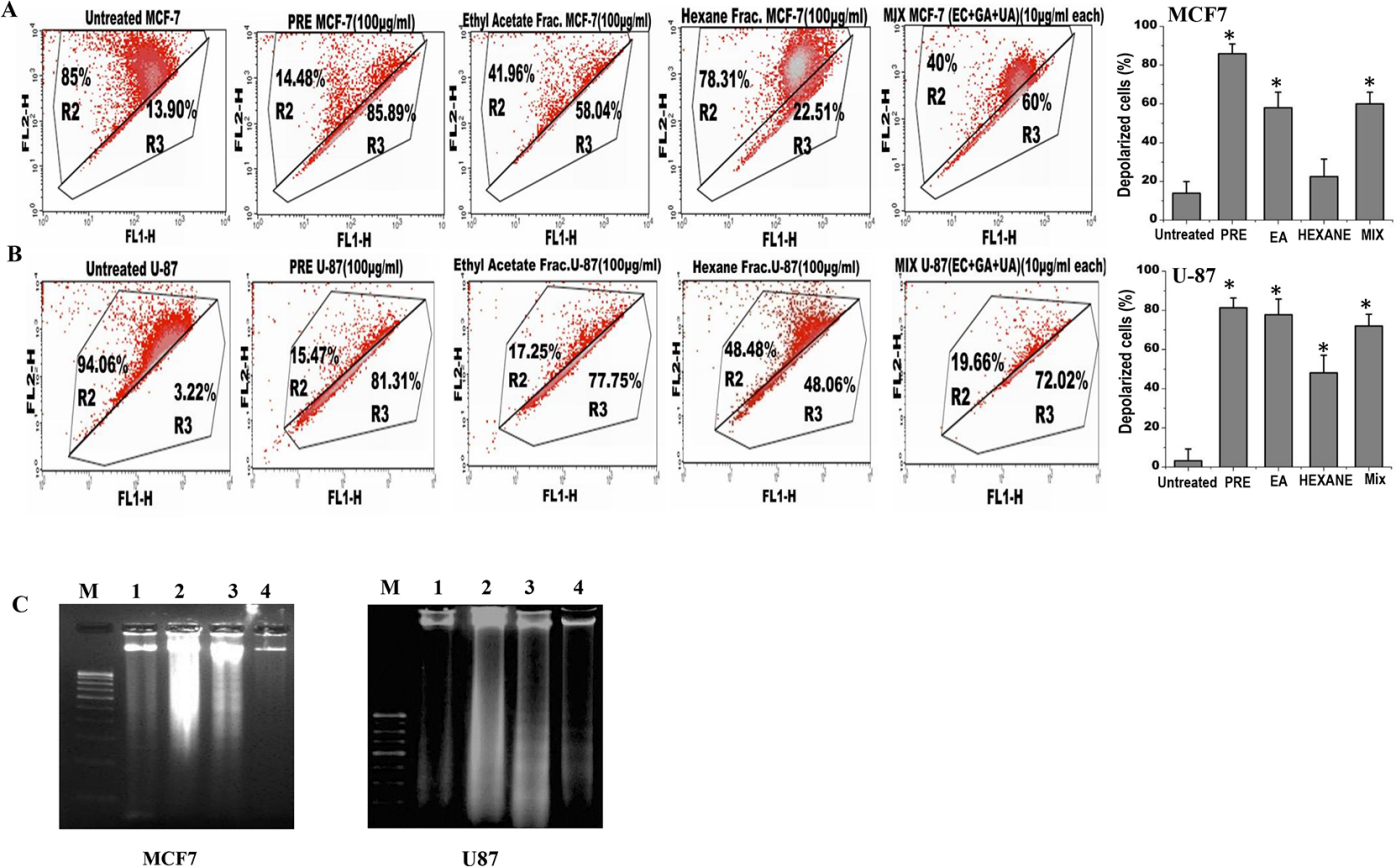
Analysis of apoptosis in (A) MCF7 and (B) U87 cells by measuring the mitochondrial membrane potential after JC1 staining in untreated and treated with PRE, EA, Hex fractions and mixture of EC+GA+UA of EA fraction of *P*. *fulgens* root. The upper part indicates the percentage of cells show polarization of mitochondrial membrane and the lower part shows the percentage of cells having mitochondrial membrane depolarization. All these experiments were repeated twice. Right panel shows percentage of depolarized cells after each treatment. *p<0.05, Students’ t-test as compared with untreated control. **(C)** Effect of PRE, Hexane and ethyl acetate soluble fraction treatment on the DNA degradation and fragmentation in MCF7 and U87 cells. DNA samples are labeled as: (M) molecular weight marker, (1) untreated cells, (2) cells treated with hexane-fraction, (3) cells treated with PRE and (4) cells treated with ethyl acetate fraction. Results are representative of two independent experiments. The concentration was used 100 μg/ml and the treatment was given for 24 h.

Analysis of the mitochondrial membrane potential by JC1 labelling revealed that percentage of polarized cells was significantly reduced in both MCF-7 and U87 cells after treatment with either PRE, EA or mixture of EC+GA+UA (Fig 4A and 4B; Fig B in S1 File). The reduction of polarized cells was lesser with Hex-fraction in both the cells. The untreated cells mostly exhibited red fluorescence, indicating an intact mitochondrial membrane potential. Upon treatment with PRE or EA or EC+GA+ UA the number of cells enhanced, as shown by green fluorescence. The right panel of the figure (Fig 4A and 4B) showed significant increase in the cells with mitochondrial membrane depolarization after the treatment with PRE, EA-fraction and the mixture of EC+GA+UA.

### DNA fragmentation analysis

To validate the induction of apoptosis by PRE, EA or mixture of EC+GA+UA in both MCF-7 and U87 cells, DNA fragmentation analysis was performed (Fig 4C). It was observed that treatment with PRE and EA-fraction (100 μg/ml) for 24 h showed DNA degradation in association with DNA laddering in U87 cells (panel 3 and 4), however in MCF-7 cells partial and complete DNA degradation was observed in lane 3 and 4, respectively without DNA laddering. DNA degradation could not be observed in these cell lines after treatment with Hexane-fraction for 24 h. Taken together, the PRE and EA-soluble fraction induced DNA degradation in both U87 and MCF-7cells which is the biochemical hallmark of apoptotic cell death.

### Detection of the PARP-1 proteolysis and expression level of apoptosis inhibitors

**Fig 5:**
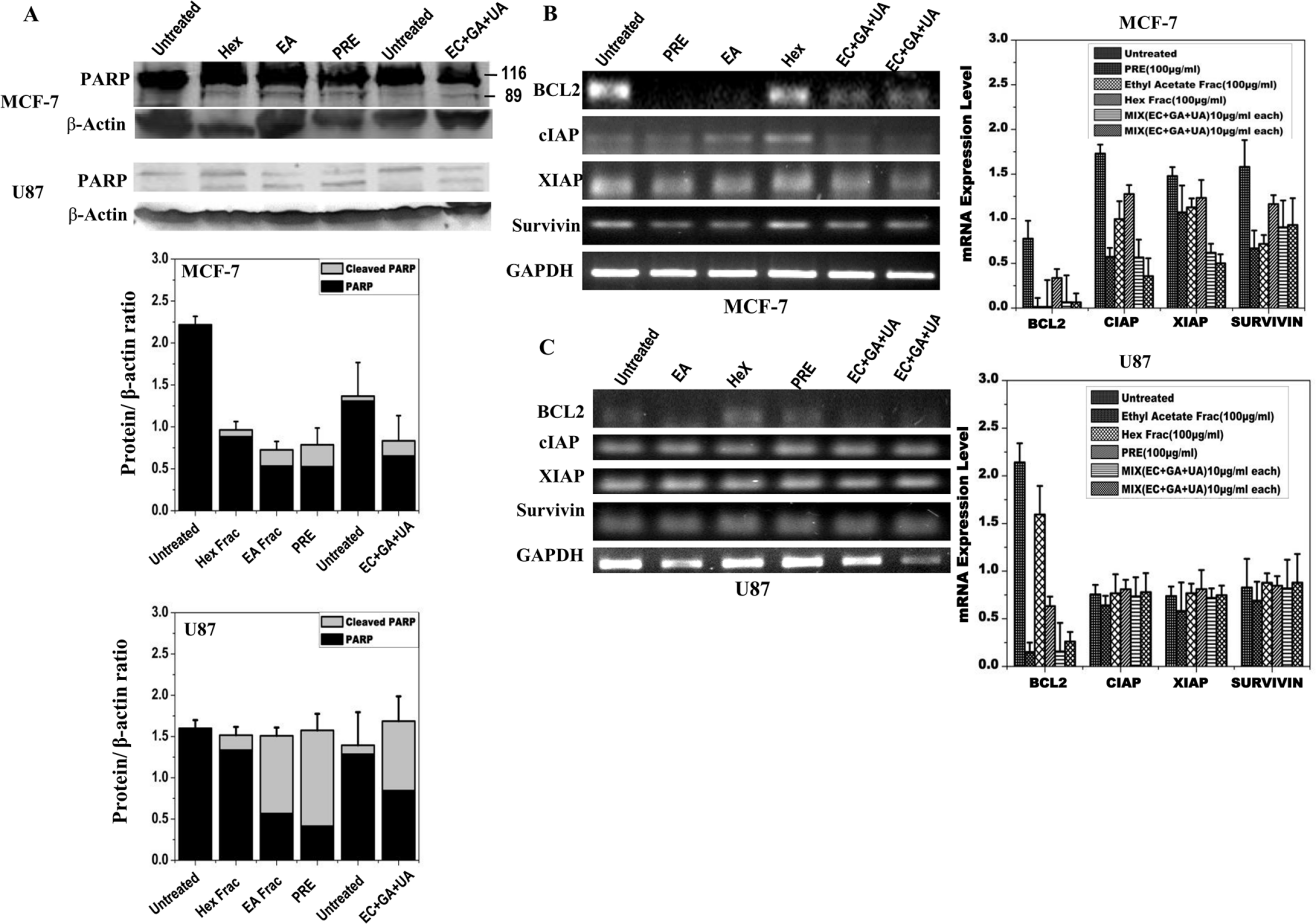
Protein expression analysis of MCF-7 and U87 cells by Western blotting after treatment with PRE, EA, Hex fractions and EC+GA+UA of EA fraction of *P*. *fulgens* root extracts in comparison with the untreated samples. **(A)** Determination of PARP cleavage. Lower panel shows quantitative densitometric analysis of the level of PARP with and without cleavage in the treated and untreated MCF-7 and U87 cells. The values are the mean±SEM of three independent experiments. Two independent untreated samples were used. The values are normalized to respective β-actin values. Expression pattern of Bcl2, survivin, XIAP and cIAP using semiquantitative RT- PCR in **(B)** MCF-7 and **(C)** U87 cells treated with or without PRE, EA, Hex fractions and EC+GA+UA (two independent samples). Right-side panel shows quantitative densitometric analysis of the expression profile of genes mRNA level. The values are the mean±SEM of two independent experiments and are normalized to respective GAPDH values.

In order to confirm that the cell death induced by PRE, EA or mixture of EC+GA+UA in both MCF-7 and U87 cells was due to apoptosis, cleavage of PARP was measured in both the cell lines after 24 h treatment (Fig 5A). PARP-1 proteolysis was detected after the treatment with PRE, EA and mixture of EC+GA+UA in both the cells (Fig 5A). Consistent with this observation, significant reduction in the expression of Bcl2 in both MCF-7 and U87 cells was also observed following treatments with PRE, EA fraction and the mix-compounds (Fig 5B and 5C). Treatment with Hex-fraction failed to reduce the level of Bcl2 in both the cell types. Besides Bcl2, the expression of inhibitors of apoptosis proteins was also reduced after these treatments in MCF-7 cells, whereas in U87 cells such reduction was not observed.

### Level of reduced glutathione (GSH) and expression level of GCLC

**Fig 6:**
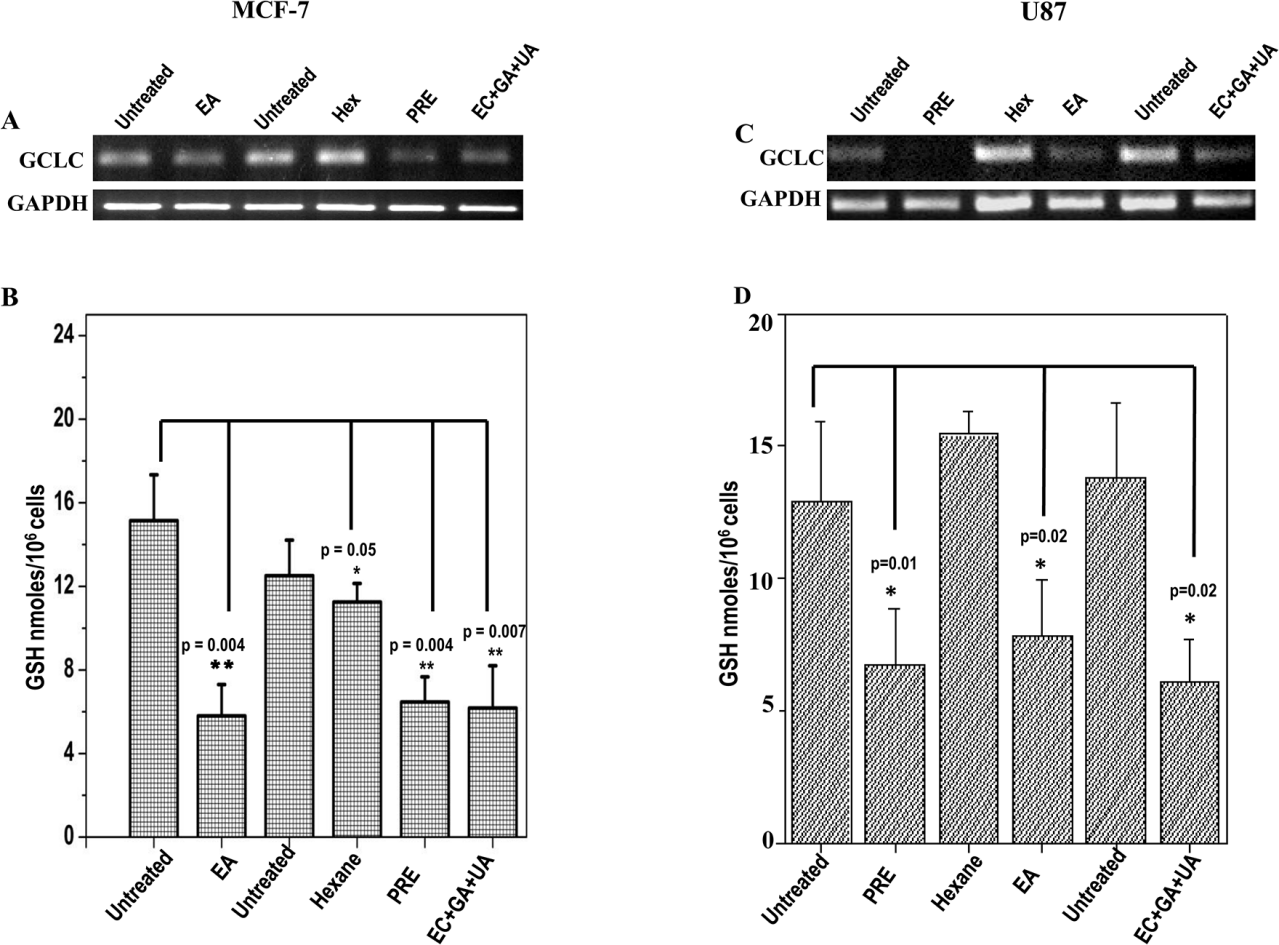
Expression pattern of GCLC in (A) MCF-7 cells and in (C) U87 cells treated with or without PRE, EA, Hex fractions and mixture of EC+GA+UA. Two independent untreated samples were used in this study. Lower panel shows quantitative densitometric analysis of the expression profile of GCLC mRNA level. The values are the mean±SEM of two independent experiments and are normalized to respective GAPDH values. Levels of glutathione in **(B)** MCF-7 cells and **(D)** U87 cells treated with or without PRE, EA, Hex fractions and EC+GA+UA. Two independent untreated samples were used in this study. Values are mean±SEM of three different experiments. *p<0.05, Students’ t-test as compared with untreated control.

Level of reduced GSH in MCF-7 and U87 cells is shown after the treatment with PRE, EA, Hex and EC+GA+UA (Fig 6B and 6D). The concentration of GSH was reduced significantly in both the cells after the treatment with PRE, EA and EC+GA+UA.

This reduction in GSH-level is accompanied by the lesser expression of GCLC in the treated MCF-7 and U87 cells (Fig 6A and 6C).

## Discussion

*P*. *fulgens* root-stock and whole herb are commonly used as folk medicine by natives of northeast India, Nepal and Bhutan for variety of ailments [8]. By using various analytical techniques with the crude extract it was shown the presence of triterpene acids and B-type flavan-3-ols in the extract which are generally known for various biological activities [11]. Solvent-solvent partitioning of methanol extract yielded hexane, ethyl-acetate, *n*butanol and aqueous extract. The present study demonstrated promising anticancer activity of the crude methanolic extract of *P*. *fulgens* root and its EA-fraction against MCF-7 and U87 cells. The concentration used in this study for PRE, EA- and Hex-fraction was 100 μg/ml, which was higher than the individual IC_50_ value. All these extracts were evaluated earlier for antioxidant and cytotoxic activities and the EA-fraction was found to be the most active [10]. In this study, it was observed that treatment with PRE or EA-fraction killed significantly more cancer cells than normal cells. Both PRE and EA-fraction caused dose-dependent reduction in the cloning efficiency in both cell lines, however such reduction in cloning-efficiency was not observed with Hexane- and *n*butanol-fractions. Since EA-fraction showed most efficient growth inhibitory effect, it was further purified and tested for their anticancer activity.

Earlier nine compounds and some monomeric and dimeric flavan-3-ols were identified and characterized from the EA-fraction of the methanolic root extract of *P*. *fulgens* [10]. From these nine compounds, three compounds viz., epicatechin (EC), gallic acid (GA) and ursolic acid (UA) were selected and mixed together. The selection of these compounds was made from three different sub-fractions and was based on their higher yield with respect to amount; GA (3.6%), UA (3.3%) and EC (2.7%) in crude methanol extract [10,11]. UA was found to be the most active compound among the isolated triterpene acids with IC_50_ 5.3 and 7.8 μM in DPPH^•^ and ABTS ^+•^ antioxidant assay [10,11].

In this study, MCF-7 cells was more sensitive than U87 cells since significant reduction in cell growth was obtained by lower concentration of PRE and EA-fraction. Such inhibition in growth of these cell lines could be attributed due to either inhibition in cell proliferation or killing the cells. The flow cytometric analysis demonstrated an increased fraction of cells in sub-G1 indicating cell death which could be due to apoptosis. To obtain additional evidence for apoptosis, we tested whether the dying cells exhibited other characteristics of programmed cell death. Flow cytometric analysis demonstrated significant reduction of polarized cells in mitochondrial membrane whereas immunoblot results showed cleaved PARP-1 protein in the samples treated with PRE, EA-fraction and the mixture of EC+GA+UA. PARP is activated at an intermediate stage of apoptosis and is inactivated by proteolytic cleavage at a late stage by caspase3 and caspase7. Such proteolytic cleavage of PARP is considered as hallmark of apoptosis [17]. Thus present results indicate that the crude extract of *P*. *fulgens* root as well as its EA-fraction induce apoptosis in these cancer cells and support the notion that some natural compounds including plants induce apoptosis that are blocked in cancer cells [18].

The apoptotic effect of PRE and EA-fraction was further confirmed by the DNA fragmentation assay. These treatment extensively degraded DNA in U87 cells, suggesting that cells treated with PRE or EA-fraction underwent apoptosis. Normally, DNA fragmentation due to DNA degradation is considered a biochemical hallmark of apoptosis that involves cutting the inter-nucleosomal regions into small fragments of double-stranded DNA [19]. Thus, changing in the DNA characteristics after the treatment with PRE, EA-fraction or the mixture of EC+GA+UA confirmed cell death induction in U87 cells. Partial and adequate DNA degradation was also noticed in the MCF-7 cell line without DNA ladder formation which could be explained by the lack of caspase-3 in this cell line [20]. It was observed that MCF-7 cells can be killed by apoptotic stimuli, such as tumour necrosis factor and staurosporine, without DNA fragmentation [20]. Therefore, the potential of cells undergoing apoptosis by the treatment with PRE, EA-fraction and EC+GA+UA cannot be ruled out in these cell lines by showing PARP-clevage with or without DNA-fragmentation.

It is an established fact that the Bcl-2 family members either inhibitor (Bcl-2) or activator (Bax), play an important regulatory role in apoptosis [21]. The present data indicate that the induction of apoptosis was due to reduction in the expression of Bcl2, an anti-apoptotic protein, in both MCF-7 and U87 cells. Similar induction of apoptosis by down-regulating Bcl-2 expression in lymphocytic leukaemia cell line CEM-C7H2 was achieved after the treatment with PADMA 28 (a herbal mixture of 28 plants including aerial part of *Potentilla aurea)* [22]. Moreover, in MCF-7 cells the induction of apoptosis was also achieved by down-regulating inhibitor of apoptosis proteins (IAPs) like cIAP, XIAP and survivin after the treatment with PRE, EA-fraction and EC+GA+UA. Such down-regulation of XIAP for the induction of apoptosis was reported earlier after the treatment with Isorhapontigenin, isolated from a Chinese herb *Gnetum cleistostachyum* [23]. Kaempferol, a common flavonoid, present in many *Potentilla sp*, enhances TRAIL-induced apoptosis by down-regulation of IAPs in glioma cells [24]. However, the downregulation of IAPs was not seen in U87 cells in this study. Thus current data provide important insights into the understanding of the molecular mechanism(s) that are responsible for the anti-cancer effect showed by PRE and its EA-fraction.

A novel aspect of this study was the analysis of the level of endogenous GSH in MCF-7 and U87 cells after the treatment. GSH is the major cellular non-protein thiol antioxidant in normal mammalian cells and in many malignant cell types. Higher level of GSH is associated with a proliferative response [25]. Usually the level of GSH in tumor tends to be higher than normal tissues and thus malignant cells show multidrug or radiation resistance [26,27]. In this study, endogenous GSH was depleted significantly in both the cells after the treatment with PRE, EA-fraction and EC+GA+UA mixture. The level of GSH was also decreased in liver and in Dalton’s lymphoma cells of PRE treated tumor-bearing mice [28]. Research over the years has generated enough evidence to implicate that the depletion of GSH is considered to be an indicator of apoptosis induction in response to a number of proapoptotic stimuli [29, 30]. Therefore, the present induction of apoptosis by EA-fraction in both the cancer cells supports the notion that depletion of GSH might favouring the apoptotic cell death. Interestingly, it was also observed in this study that such depletion of GSH in these cell lines was achieved by downregulating the expression of γ-glutamyl-cysteine synthetase heavy/catalytic subunit (GCSC). This enzyme involves in GSH synthesis [31] and consists of a catalytic subunit performs enzyme activity and a modifier subunit that modulates GCSC activity [32]. It was demonstrated earlier that quercetin and kaempferol increased the production of GSH in neuronal cells [33] and in COS-1 cells by increasing the expression of GCSC [34]. Therefore, natural plant products mediated GSH depletion or elevation by upregulating or downregulating GCSC is an important observation because earlier attempt to deplete the level of GSH by synthetic molecules have been limited due to its non-selective effects [35]. Moreover, it was noticed that those patients having higher level of GSH showed higher resistant to therapy and also managed to sustain the GSH-level than those lacking the synthetic ability [26].

It is important to note that the mixture of three compounds in this study showed similar potentiality in apoptotic induction and GSH depletion in cancer cells as it was observed with the treatment with PRE or EA-fraction. Inhibition of carcinogenesis and invasiveness in animal models and in vitro cancerous cell lines was demonstrated earlier by a polyphenol gallic acid [36, 37]. (-)- Epicatechin exhibits several beneficial effects to human health [38] and sensitizes several cancer cells towards radiation [39]. A pentacyclic triterpene Ursolic acid has been shown to have the effects of anti-inflammatory, antioxidant, and antitumor [40,41]. Through elevation of intracellular calcium ion [42] and upregulating of death receptors [43] Ursolic acid can induce apoptosis in tumor cell. Therefore, all these compounds show anticancer potentialities with different targets. Thus pharmaceutical companies have been looking for developing multitargeted therapies by either combining several monotargeted drugs or to develop drugs having multitargeting properties. It was observed that several plant-derived dietary agents, have multitargeting properties and some such compounds have already been approved for human use [44]. In this study, the observed modulation in the level of GSH-concentration by the EA-soluble fraction of the *P*. *fulgens* root extract in tumor cells opened up the possibility of a new therapeutic approach because these plant products are not harmful to normal cells and also regulating the tumor cellular response to different anticancer treatments [45]. Therefore, it would be interesting to examine efficacy of these plant products in human cancer treatment.

## Supporting Information

S1 File Extended experimental procedures. Flow cytometric analysis of cell death by PRE and EA-fraction in normal and cancer cells. **Fig A**. The flow cytometry analysis on cell cycle distribution of PI-labeled cells in human lymphocytes, MCF-7 cells and U87 cells with and without treatment with PRE, EA-fraction of *P*. *fulgens* root extract. **Fig B.** Histogram Plot showing Red fluorescent JC-1 aggregates (FL2-H) in MCF-7 & U-87 cells. Histograms showed decreased florescence intensity depicted by peak shift (M2) between untreated samples and treated samples indicating a decrease in the Mitochondrial membrane potential in the treated samples (Total 10000 cells were acquired for each sample). **Table A.** Primer sequences and the product sizes.
(DOCX)
