# Supplementary material for: Induction of Apoptosis and Reduction of Endogenous Glutathione Level by the Ethyl-Acetate Soluble Fraction of the Methanol Extract of the Roots of Potentilla fulgens in Cancer Cells
Source: PLoS One. 2015 Aug 18;10(8):e0135890. doi: 10.1371/journal.pone.0135890 (PMC4540452; doi:10.1371/journal.pone.0135890)
Supplement: S1 File — Flow cytometric analysis of cell death by PRE and EA-fraction in normal and cancer cells. Fig A. The flow cytometry analysis on cell cycle distribution of PI-labeled cells in human lymphocytes, MCF-7 cells and U87 cells with and without treatment with PRE, EA-fraction of P. fulgens root extract. Fig B. Histogram Plot showing Red fluorescent JC-1 aggregates (FL2-H) in MCF-7 & U-87 cells. Histograms showed decreased florescence intensity depicted by peak shift (M2) between untreated samples and treated samples indicating a decrease in the Mitochondrial membrane potential in the treated samples (Total 10000 cells were acquired for each sample). Table A. Primer sequences and the product sizes. (DOCX) [file pone.0135890.s001.docx]

**Supporting Information**

**Extended experimental procedures**

*Flow cytometric analysis of cell death by PRE and EA-fraction in normal and cancer cells*

PHA-stimulated (24 h after PHA addition) lymphocytes were grown in culture for 24 h with and without PRE or EA-fraction (100 µg/ml) and then fixed with 70% ethanol. In case of MCF-7 and U87cells, cells were fixed with 70% ethanol soon after the treatment for 24 h.

The fixed cells were washed in PBS and resuspended in 500 µl of propidium iodide solution (50 µg/ml propidium iodide, 0.2 mg/ml RNase) for 1 h at room temperature in dark. 10,000 cells were acquired for each sample and analysed with a FACS Calibur (Becton-Dickinson). CELLQuest Pro software was used to quantify cell cycle compartments to estimate the percentage of cells distributed in the different cell cycle phases.

## **Results**

*Flow cytometric analysis*

Flow cytometric analysis (Figure A) after PRE and EA-fraction treatment showed reduction in G1 cells and increase in sub-G1 cells in cancer cell lines. However, for human lymphocytes, the increase in the frequency of sub-G1 was marginal in the PRE and EA treated samples.

**Figure A**. The flow cytometry analysis on cell cycle distribution of PI-labeled cells in human lymphocytes, MCF-7 cells and U87 cells with and without treatment with PRE, EA-fraction of *P. fulgens* root extract.


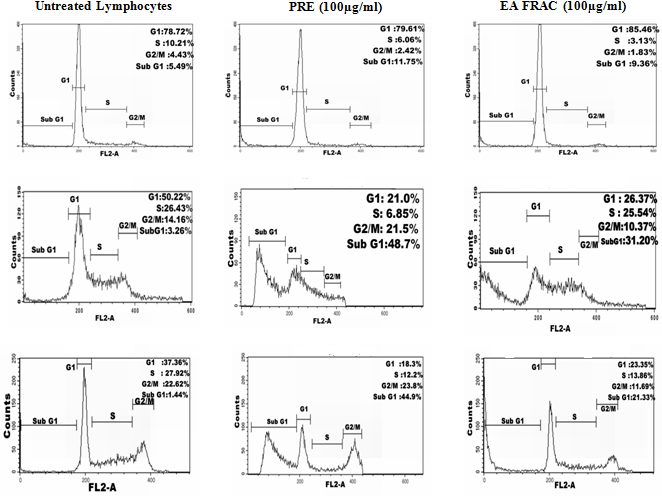


**Untreated MCF-7**

**Untreated U87**


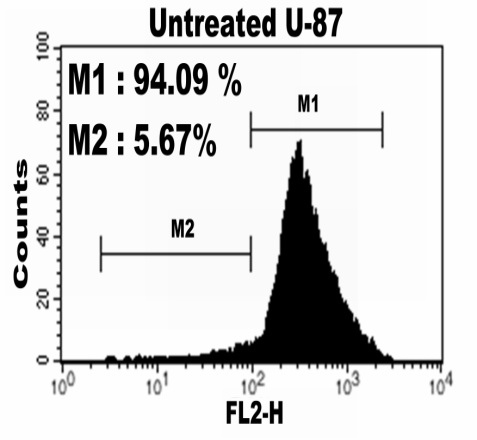

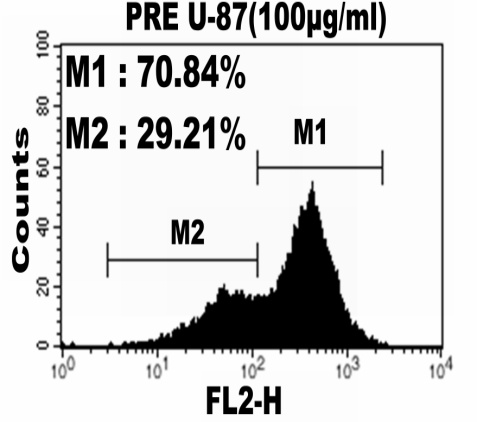

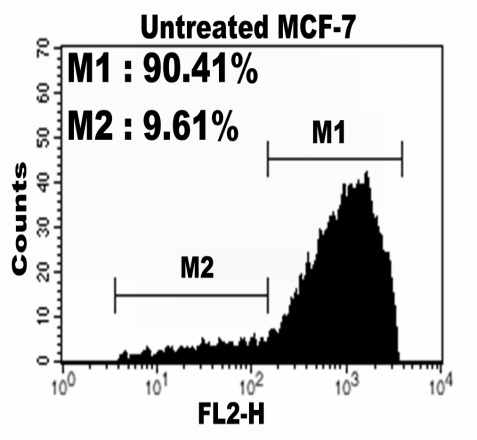

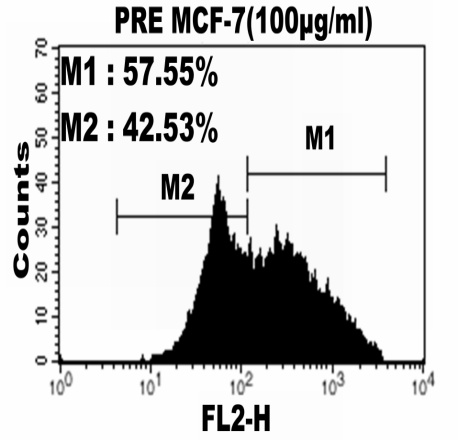


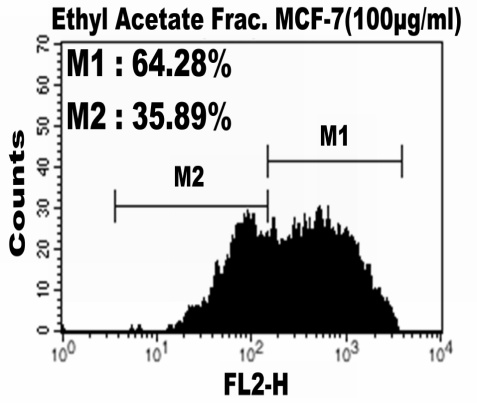

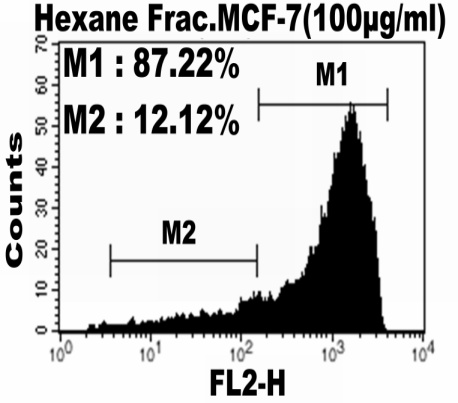

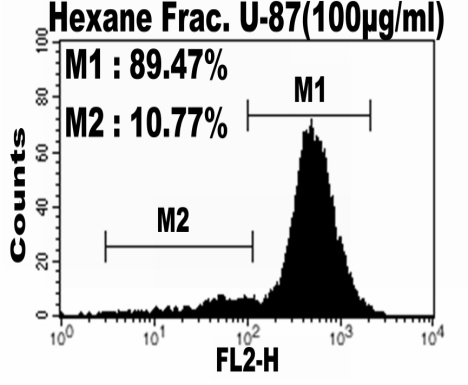

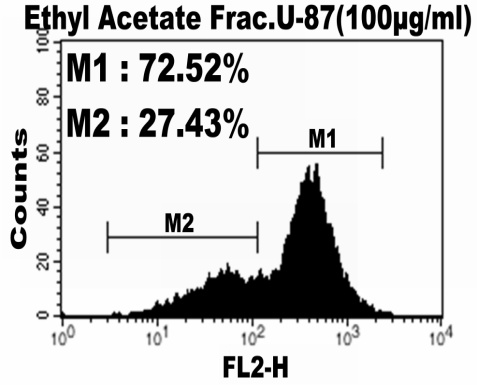


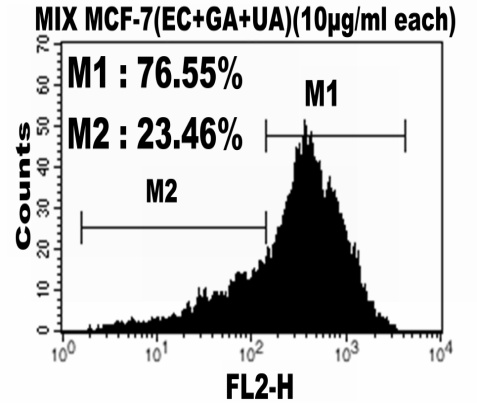


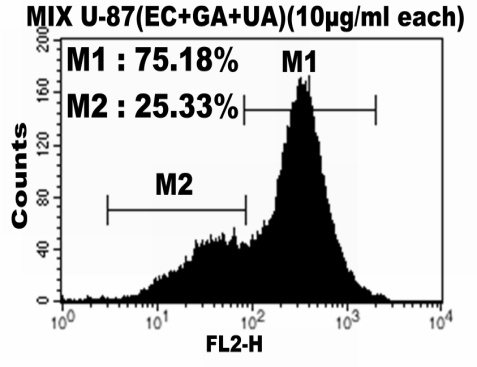


**Figure B** Histogram Plot showing Red fluorescent JC-1 aggregates (FL2-H) in MCF-7 & U-87 cells. Histograms showed decreased florescence intensity depicted by peak shift (M2) between untreated samples and treated samples indicating a decrease in the Mitochondrial membrane potential (∆ψm) in the treated samples (Total 10000 cells were acquired for each sample).

**Table A: Primer sequences and the product sizes**

| **Sl No** | **Oligo name** | **Sequence** | **Product Size** |
| --- | --- | --- | --- |
| 1 | **Bcl2 (F)** | **ACATCGCCCTGTGGATGACT** | **206bp** |
|  | **Bcl2 (R)** | **TCACTTGTGGCCCAGATAGG** |  |
| 2 | **Ciap1 (F)** | **TGTTGTCAACTTCAGATACCACTGG** | **98bp** |
|  | **Ciap1 (R)** | **CATCATGACAGCTCTTCTGAAGA** |  |
| 3 | **XIAP (F)** | **GACAGTATGCAAGATGAGTCAAGTCA** | **93bp** |
|  | **XIAP (R)** | **GCAAAGCTTCTCCT CTTGCAG** |  |
| 4 | **Survivin (F)** | **AGTGAGGGAGGAAGAAGGCA** | **80bp** |
|  | **Survivin (R)** | **ATTCACTGTGGAAGGCTCTGC** |  |
| 5 | **GCLC (F)** | **GGAAGGAAGGTGTGTTTCCTGG** | **162bp** |
|  | **GCLC (R)** | **ACTCCCTCATCCATCTGGCAA** |  |
| 6 | **GAPDH (F)** | **ACAGTCCATGCCATCACTGCC** | **265bp** |
|  | **GAPDH (R)** | **GCCTGCTTCACCACCTTCTTG** |  |
